# Energy consumption and thermal comfort of rock-cut and modern buildings

**DOI:** 10.1016/j.heliyon.2024.e34217

**Published:** 2024-07-08

**Authors:** Mohammad Mangeli, Farshid Aram, Reza Abouei

**Affiliations:** a Faculty of Architecture, Urbanism, and Art, Urmia University, Urmia, 5756151818, Iran; b Faculty of Art and Architecture, Yazd University, Yazd, 8915818411, Iran

**Keywords:** Indoor thermal comfort, Energy consumption, Rock-cut architecture, Meymand, Vernacular architecture

## Abstract

Energy consumption in the building sector justifies the necessity of knowing the thermal comfort perception of vernacular and modern architectural types, based on which a correct recognition was reached for the design of buildings suitable for the climatic conditions of each region. It should be determined that the different types of modern and traditional architecture are in the comfort level in harsh hot climate conditions and how much energy they consume to reach the comfort level. Despite consideration of energy consumption and thermal comfort in different buildings in Iran, there is no clear framework for evaluating these two parameters in different buildings and comparing them. This research aims to compare the indoor thermal comfort levels of vernacular architectural buildings and modern buildings in Iran's semi-hot and dry climate at the peak of summer heat and determine their energy consumption to reach the comfort level. This study has been accomplished by collecting field data, examining the indoor predicted mean vote (PMV) index of the buildings, and comparing them. It was found that rock-cut architecture buildings are in better thermal comfort conditions without energy consumption due to the use of groundmass temperature and low heat exchanges between the indoors and outdoors because of the thermal phase of the materials and the thickness of its layers. The indoor PMV average of rock-cut buildings in summer is −0.61; in modern buildings, it is 0.77, while these two building complexes are in the same climate and close. Also, the energy consumption to reach the comfort level in rock-cut buildings is zero, while modern buildings consume an average of 7.7 kW of electricity daily. The research results will lead to recognizing and modeling the climate design of vernacular architecture, which can be used in today's architecture to reduce energy consumption.

## Highlights

• Comparison of thermal comfort and energy consumption in vernacular and modern buildings.
• Rock-cut architecture provides better thermal comfort without energy consumption.
• Modern buildings consume an average of 7.7 KW of electricity daily to reach comfort level.
• Indoor PMV average: −0.61 in rock-cut buildings, and 0.77 in modern buildings.

**Table:** Nomenclature

°C	Degree Celsius	Blg.	Building
df	Degrees of Freedom	CBE	Centre for Built Environment
F	Fisher test value	Clo	Clothing Level
H·P	Horsepower	sig	Significance level
K	Kelvin	ISO	International Organization for Standardization
kJ	Kilojoules	Met	Metabolism
p	p-value	PMV	Predicted Mean Vote
R.H	Relative Humidity	PPD	predicted percentage of dissatisfied
U	Mann–Whitney *U* test level	SET	Standard Effective Temperature
$\bar{Z}$	Z-test value	STD	Standard
**Abbreviation**		UNESCO	United Nations Educational, Scientific and Cultural Organization
ASHRAE	American Society of Heating, Refrigerating and Air-Conditioning Engineers		

## 1 Introduction

Understanding the climatic perceptions and thermal comfort in historical buildings and complexes eases the attainment of innovative design patterns and architectural critiques, aiming to reduce energy consumption in contemporary structures [1,2]. Comparing the thermal comfort perceptions of historical buildings to modern structures contributes to a better comprehension of architecturally suitable conditions for both architectural genres in varying climates [[3], [4], [5]]. The Vernacular architectural buildings, due to their harmonious alignment with each region's climate and geographical context encapsulate architectural experiences. The harmonized propose is creating environmental comfort with minimal facilities by addressing unfavorable environment and weather conditions using natural resources and architectural features. The lessons learned from vernacular architecture can be applied in contemporary architecture to reduce energy consumption and achieve sustainability [6,7] These features encompass heating through mass, thickness of building layers, use of materials with high thermal conductivity, orientation, spatial depth, phase change of materials, and utilization of natural light and ventilation. These elements, which are present in historical and vernacular architectural structures, can contribute to energy efficiency within a passive system [[8], [9], [10]]. Rock-cut architecture, a subtype of vernacular architecture, utilizes subterranean mass heating to create a space with minimal temperature fluctuations [[11], [12], [13]]. Throughout history, the structures of this architectural style have found broader application in harsh climates such as warm and arid or cold regions. The reason for this prevalence is not only the ease of execution and technical feasibility but also the minimal energy usage required to adapt the structure to thermal comfort conditions [14,15]Therefore, comparing the internal thermal comfort and energy consumption of samples from this architecture in warm and arid climates with samples from modern architecture provides a better understanding of the climatic performance of both styles.

Relevant research has primarily focused on evaluating the level of thermal comfort experienced by residents of traditional buildings, comparing it with the comfort levels in modern residential structures [[16], [17], [18]] Moreover, these studies have highlighted factors influencing the differences in thermal comfort levels between modern and traditional buildings. Some studies have specifically targeted the evaluation of architectural modifications, such as adding pillars or thermal insulation layers and altering the orientation of the structure [19,20]. In some studies, the assessment of the thermal comfort level of residents in both traditional and modern buildings is evident. These studies have demonstrated that inhabitants of traditional structures experience a lower expected level of thermal comfort compared to residents of modern buildings, particularly in summer where traditional buildings better meet their occupants' thermal comfort expectations. Conversely, this trend tends to reverse in winter [17,18,21,22]. In Iran, most of the studies have focused on assessing the thermal comfort of residents in traditional buildings across various climates by employing questionnaires and comparing them with the experiences of users in different structures. The findings suggest that occupants of traditional buildings experience higher levels of comfort during warm seasons compared to the residents of modern buildings in the same conditions [[23], [24], [25], [26]]. Furthermore, some have focused on modeling influential factors on thermal comfort, such as natural ventilation and structure orientation, based on ASHRAE standards in traditional buildings across different climates. The results indicated that these buildings exhibit more suitable thermal perceptions during warm seasons [27,28].

In the researches conducted so far in Iran, the comparison of thermal comfort and energy consumption in certain types of vernacular architecture with contemporary architecture has not been discussed and there is a necessity to conduct this type of research. Also, in these researches, no specific framework has been mentioned in the field of how and the form of this comparison that compares thermal comfort in vernacular buildings with modern buildings until now. One of the existing deficiencies and issues is the lack of a specific research framework for comparison in the energy consumption and thermal comfort fields. This research tries to provide a basic framework for this form of comparison even though similar research about developing a research framework for this field in Iran has not been an issue so far, also the issue of energy consumption and thermal comfort in vernacular and historic buildings has been less considered. Furthermore, the investigation and comparison of the thermal behavior of these two types of buildings during the peak summer heat, when the highest amount of energy consumption is needed to bring the building to the level of thermal comfort, is not seen. Therefore, with the lack of knowledge in the field of energy consumption in traditional buildings during peak consumption times, the possibility of judging the level of sustainability of these buildings is limited. Consequently, this essential question is raised whether vernacular buildings have better thermal comfort levels in the hot season than contemporary buildings and provide comfortable conditions for the residents with less energy consumption. It is also hypothesized that rock-cut architectural buildings, as a type of Iranian vernacular architecture, have better comfort conditions in the hot season than modern buildings, without energy consumption. Thus, the present study aims to simultaneously compare the indoor thermal comfort levels of rock-cut vernacular architectural structures and modern buildings using the predicted mean vote (PMV) index in the semi-hot arid climatic zone of Iran during the peak summer heat to determine which category of buildings is closer to internal thermal comfort conditions. Introducing and studying an example of vernacular architecture that provides thermal comfort without consuming energy in the heat of summer, in hot arid and semi-hot arid climates, will be done in this research to be able to take advantage of its architectural features for use in modern climate architecture design and approaching sustainability in architecture through reducing energy consumption. It should also be mentioned that the case study of the UNESCO World Heritage complex of Meymand is a unique pattern of Iranian vernacular architecture that introduces a model of sustainable architecture that continues to live there after centuries. Comparing with the modern architecture of the region can lead to a better understanding of this valuable architectural heritage of Iran and the conservation and aspects of its climate sustainability.

## 2 Methodology

The present study employed a field survey approach to collect data related to temperature, R.H, and air velocity for the analysis and comparison of internal thermal comfort levels in rock-cut vernacular architectural structures and a city with modern architecture. The required data for analyzing thermal comfort during the summer months, from July to September 2022, were collected weekly at both locations. Simultaneously, these data were collected in the outdoor ambient air at both locations for measuring the indoor and outdoor PMV levels. During the data collection, the buildings within the UNESCO World Heritage site of Rock-cut Architecture in Meymand did not utilize any cooling devices. However, in Shahr-e Babak, a city with modern architecture located near the UNESCO World Heritage site of Rock-cut Architecture in Meymand, the buildings in this city used evaporative coolers for cooling purposes.

### 2.1 Area of the study

#### 2.1.1 Meymand

**Fig. 1:**
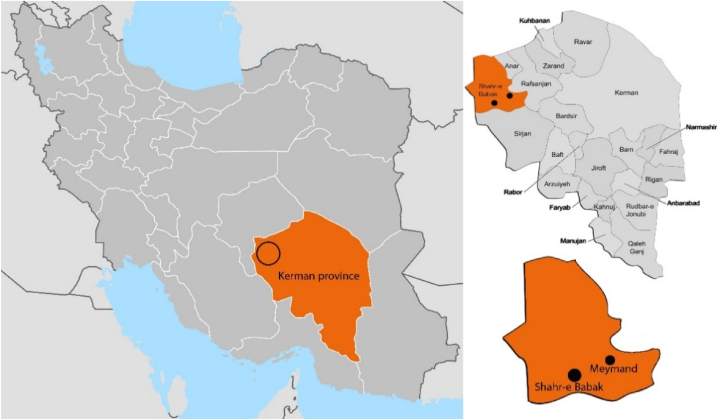
Meymand and Shahr-e Babak location in Iran's map.

The UNESCO World Heritage Site of Rock-cut Architecture in Meymand encompasses more than 370 rock-cut architectural units. All these structures are crafted by excavating architectural spaces within pyroclastic rock masses, primarily composed of Pumice and Tuff deposits. Situated at an elevation of 2250 m above sea level and located at coordinates 30° 15′ North and 55° 22′ East in central Iran (Fig. 1). The historical origins of this architectural complex date back to the Parthian and Sassanian periods (250 BCE) [14,15]. Since then, the indigenous people have continued to inhabit this site. As for 2012, UNESCO statistics indicate that around 300 individuals reside in this historical complex [29]. Climatically, this complex is situated in a semi-warm and dry mountainous region [30,31]. The region experiences significant day-night temperature variations throughout the year, characterized by cold winters and hot, dry summers due to low humidity and sparse vegetation coverage.

#### 2.1.2 Shahr-e Babak

For the necessary comparison in this research, buildings of modern architecture of Shahre Babak, a city near Meymand, were studied because this city is the closest place to Meymand, which has modern architecture and is in a similar geographical and climatical zone. This city is situated in the northwest of Kerman province, in central Iran, 30 km from Meymand. Shahr-e Babak's elevation above sea level is 1850 m, positioned at coordinates 30° 11′ North and 55° 12′ East (Fig. 1). Climatically, the city is in a semi-warm and dry mountainous region [30,31], experiencing cold winters and hot, dry summers, like Meymand. The annual average rainfall of the city is approximately 150 mm, contributing to the feverish temperature difference between day and night due to limited precipitation and humidity [32]. Most buildings in this city have a lifespan of less than 30 years and have been constructed with modern design using concrete and brick materials.

### 2.2 Selected buildings for monitoring in Meymand

**Fig. 2:**
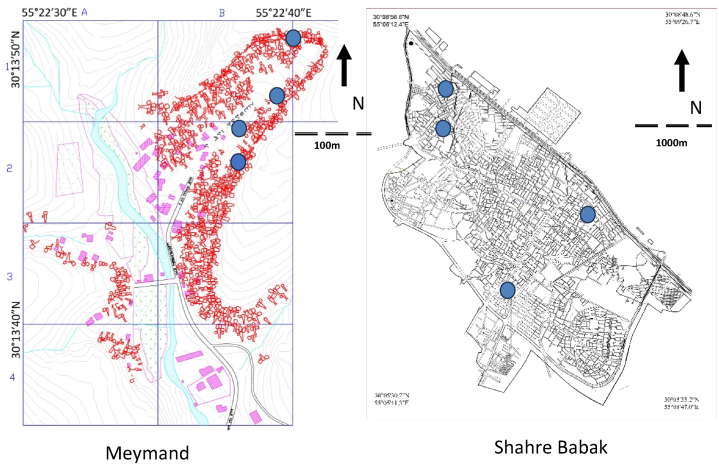
Meymand and Shahre Babak general map and selected studied building's location.

**Fig. 3:**
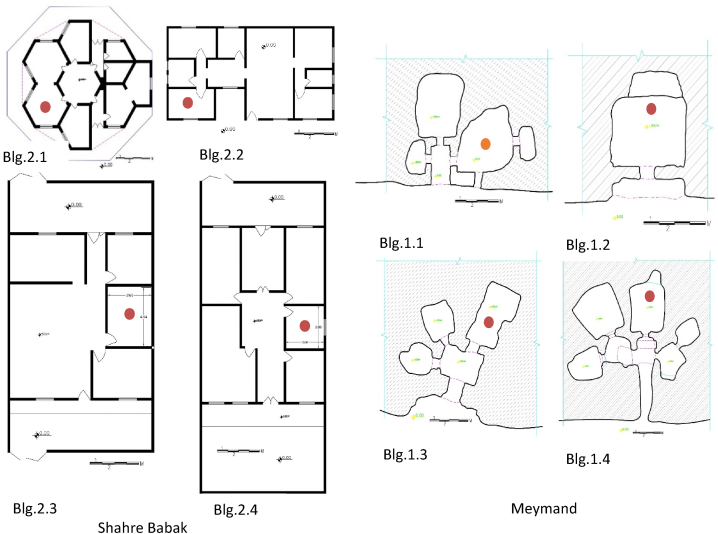
Selected buildings plans and installation places in Meymand and Shahre Babak.

To collect the data, four residential buildings in various locations within this traditional architectural complex were chosen for monitoring (Fig. 2). In these selected Meymand buildings, the main room where most residents' daily activities take place was monitored (Fig. 3). Two of these four units have been excavated in the vertical stone area of the hill, The other two are horizontally excavated on the sloping hill surface. The average area of the selected Meymand buildings is 55 m^2^, with the average area of four rooms chosen for data collection being 23.7 m^2^. The walls of these buildings have considerable thickness with a minimum thickness of about 70 cm (Table 1). These buildings are separated by wooden doors with an average area of 1.1 m^2^, providing semi-open and separate spaces. They lack windows, and these doors serve as the sole means of natural ventilation and light for these structures. During the summer, despite the high outdoor temperatures during the day, no cooling appliances are used in these buildings. They remain cool without energy consumption. All four buildings have been constructed from a cohesive volcanic rock mass made of Pumice and Tuff materials, with a thermal conductivity of 0.17–0.4 W/m.K and a density of 650–1250 kg/m^3^ [33].

**Table 1:** Selected buildings properties in Meymand and Shahre Babak.

Location	Building Name	Main Material	H W/m.K	Width (m)	Height (m)	Altitude (m)	Window area (m^2^)	Door area (m^2^)	Outside wall thickness (m)	Inside wall thickness (m)	Roof thickness (m)	Area (m^2^)	Orientation	Shading (m)
**Meymand**	Blg1.1	Pumice and Tuff	0.17–0.4	5	2	2265	0	1.15	1.2	0.7	3.5	21	+180	1.2
	Blg1.2	Pumice and Tuff	0.17–0.4	4.8	1.9	2252	0	1.25	0.7	1.3	0.9	18.5	+175	0.5
	Blg1.3	Pumice and Tuff	0.17–0.4	4.9	2.2	2255	0	1.1	1.3	1.1	0.8	32.2	−30	3.1
	Blg1.4	Pumice and Tuff	0.17–0.4	4.7	2.1	2267	0	1.35	1	0.9	3	23.1	−10	0.7
**Shahre Babak**	Blg2.1	Brick	1.04	4.3	2.8	1840	2.25	2.4	0.3	0.3	0.3	16.5	--30	0.5
	Blg2.2	Concrete	1.6–2 1.1	3	2.9	1840	1.4	2.2	0.2	0.2	0.4	14	−25	0.2
	Blg2.3	Brick	1.04	3.6	2.9	1855	0	2.2	0.2	0.2	0.3	14.4	−25	0
	Blg2.4	Brick	1.04	3.6	2.8	1830	1.5	2.3	0.3	0.3	0.3	16.5	−45	0.2

### 2.3 Selected buildings for monitoring in Shahr-e Babak

Four single-story residential buildings in Shahr-e Babak have been chosen to compare thermal comfort levels with those in Meymand (Fig. 2). One of the bedrooms in each of these four buildings was monitored (Fig. 3). Constructed within the last 30 years, all four buildings adhere to the contemporary architectural style of the region. The primary construction materials used include brick, lightweight concrete blocks, cement mortar, and bastard mortar. The structure of these buildings consists of reinforced masonry materials with a concrete frame and steel. The average area of the selected houses in Shahr-e Babak is 110 m^2^. The average area of the four rooms chosen for data collection is 13.5 m^2^, and the average thickness of the external walls of these buildings is 30 cm. No type of thermal insulation has been used in these building's construction. The kind of ceiling cover is an iron beam with a cross-sectional beam. The average area of the entrance doors to the rooms is 1.9 m^2^, and the ceiling height is 2.8 m. The window area is 1.5 m^2^ (Table 1). For cooling in the summer, an evaporative cooler system is utilized, with each consuming an average of 3060 kJ (Table 2).

**Table 2:** Air conditioner properties used in Shahre Babak selected buildings.

Building	Air-conditioning System	Brand and Model	Electric motor power (HP)	Electric motor average consumption (kJ)
Blg2.1	Evaporative cooler	Absal AC70	3/4	3204
Blg2.2	Evaporative cooler	Sepehr Eletric SE600UD	1/2	2923
Blg2.3	Evaporative cooler	Absal AC70	3/4	3204
Blg2.4	Evaporative cooler	Energy EC0700	3/4	2988

### 2.4 Data collection process

**Fig. 4:**
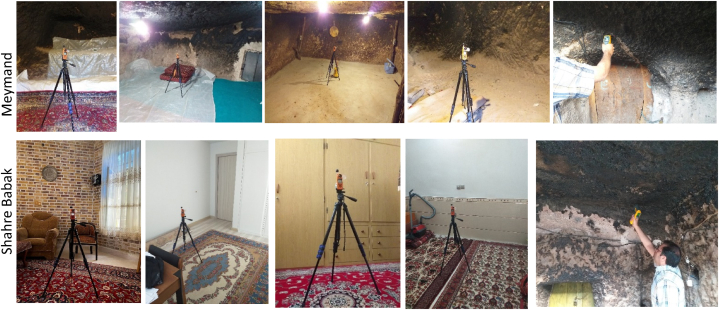
Selected rooms view and monitoring device installation and surfaces data collection in Meymand and Shahre Babak.

First, eight devices measuring temperature, R.H, and air velocity were installed within four rooms in Meymand and four bedrooms in four buildings in Shahr-e Babak. Simultaneously with this data collection, the building's indoor surface temperature in both places, including the temperature of the walls, floor, ceiling, and window, was prepared and recorded by a laser thermometer (Fig. 3). The specifications and properties of the devices are presented in Table 3. Data were simultaneously collected from mid-July to early September 2022, for seven days, at 3-Hours intervals. Monitoring devices were placed in the middle of each room, 1.10 m above the floor, and 1.5 m from doors and windows, in locations not exposed to the airflow of the air conditioning system or wind, according to the ASHRAE standard [34](Fig. 4).

**Table 3:** Monitoring device specifications and accuracy.

Measured Variables	Brand and Model	Specification Range	Resolution	Accuracy
Indoor Air Temperature	Kestrel 5400 HST	−29° to 70 °C	0.1 °C	±0.5 °C
Indoor R.H	Kestrel 5400 HST	10 %–90 %	0.1 %	±2 %
Outdoor Air Temperature	Kestrel 5400 HST	−29° to 70 °C	0.1 °C	±0.5 °C
Outdoor R.H	Kestrel 5400 HST	10 %–90 %	0.1 %	±2 %
Outdoor and Indoor Air Flow Speed	Kestrel 5400 HST	0.4–40 m/s	0.1 m/s	20 ft/min 0r 3 % larger of reading
Indoor surfaces temperature	Fluke 561 infrared thermometer	−40° to 550 °C	0.1 °C	±0.1 °C

Simultaneously, two devices were installed outdoors in Meymand and Shahr-e Babak to measure temperature, R.H, and wind speed. These devices were placed in open spaces under shade at a height of 1.70 m from the ground, and 4 m away from the building to obtain external data for PMV measurement. During data recording, the rooms were unoccupied, and no cooling system was used in Meymand. However, evaporating cooling systems have been used in all four buildings to achieve a comfort level in Shahr-e Babak. Table 4 shows the average collected data for indoor and outdoor environments.

**Table 4:** Data collection of indoor and outdoor air temperature, R.H., and airspeed in buildings.

Month	Location	Indoor		Outdoor		
		Air temperature (°C)	R.H (%)	Air temperature (°C)	R.H (%)	Wind speed (m/s)
July	Meymand	23.61	25.42	27.66	41.69	3.01
August		23.15	26.65	25.53	18.25	4.29
September		22.62	24.58	21.10	19.25	3.34
Average		23.12	25.55	24.76	26.39	3.54
Standard Deviation		0.40	0.85	2.73	10.82	0.54
July	Shahre Babak	28.73	38.80	28.34	51.88	3.87
August		27.91	21.31	27.42	15.27	4.44
September		26.57	22.20	23.40	18.13	1.84
Average		27.73	27.43	26.38	28.42	3.38
Standard Deviation		0.89	8.04	2.14	16.62	1.11

### 2.5 Data analysis

To measure the indoor PMV levels in buildings, the CBE Thermal Comfort Tool has been employed by using the data collected within the structures. This online tool follows ASHRAE and ISO standards developed by the University of California, Berkeley [35]. It utilizes environmental data such as temperature, R.H, air velocity, body coverage, and physical activity to compute indices like PMV, SET, and PPD. In addition, it has been applied in different studies to assess indoor thermal comfort [36,37].

The Rayman tool has been utilized to measure the outdoor PMV of the studied buildings in Meymand and Shahr-e Babak. Like the CBE Thermal Comfort Tool, it can measure various comfort indices for outdoor spaces and the surrounding environment. The parameters required for calculating outdoor thermal comfort indices in this software include temperature, R.H, wind speed, clothing coverage, physical activity level, and sky view factor [38,39]. In both tools, the clothing coverage was assumed to be summer casual wear with clo = 0.5, the physical activity level has been considered as sedentary with a mild activity of Met = 1.2, and a constant indoor air velocity of 0.1 m/s had been assumed for measuring indoor PMV due to the low indoor airspeed in the building. The average height and weight of residents in these buildings used for PMV calculations were 172 cm and 71 kg, respectively [29]. In this study, for comparing indoor and outdoor PMV levels, the daily PMV has been measured from 9 a.m. to 6 p.m., as the time range is crucial due to the highest temperatures and energy consumption during the day by emphasizing the significance of understanding thermal perception and comfort.

## 3 Results

### 3.1 Evaluation of outdoor environmental parameters

**Fig. 5:**
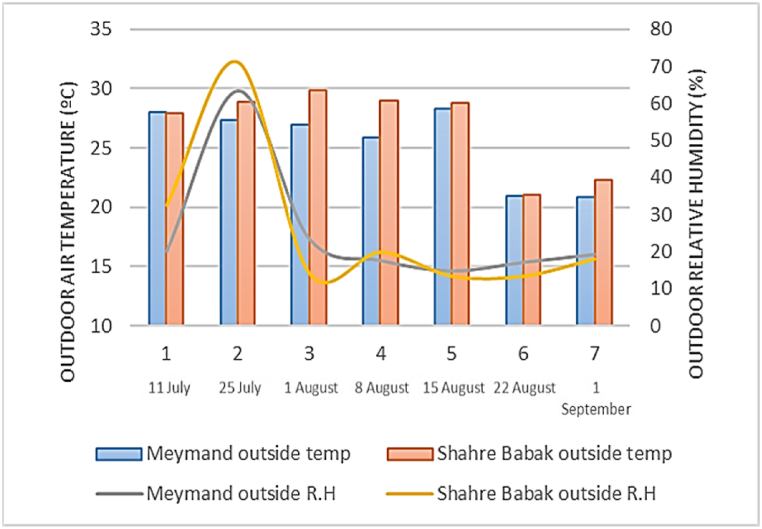
Outdoor temperature and R.H in Meymand and Shahre Babak.

Fig. 5 and Table 4 indicate the assessed outdoor environmental parameters in Meymand and Shahr-e Babak during the data collection period in the summer of 2022. Based on the data, the average outdoor air temperature in Meymand from 9 a.m.; to 6 p.m.; in summer is 24.73 °C with a standard deviation of 2.73, a recorded minimum temperature of 12.20 °C, and a maximum of 35.80 °C. Simultaneously, the average outdoor air temperature in Shahr-e Babak during the same time range is 26.38 °C with a std deviation of 2.14, a minimum temperature of 13.5 °C, and a maximum of 38.7 °C. Additionally, the R.H in Meymand was calculated at 26.39 %, with a std deviation of 10.82 and fluctuation between 11 and 81 %. In Shahr-e Babak, this parameter is averaged at 28.42 %, with a std deviation of 16.62 and fluctuation between 9 and 80 %. The average outdoor air velocity in Meymand is 3.45 m/s with a std deviation of 0.54, and in Shahr-e Babak, it is recorded at 3.38 m/s with a std deviation of 1.11. Average temperatures, humidity, and air velocity between Meymand and Shahr-e Babak were compared using an independent sample *t*-test due to the external climatic parameters during the day within the 9 a.m.; to 6 p.m.; time limit in the summer season in PMV calculation. The evaluation of the normal distribution of outdoor temperature data showed a normal distribution with a (p-value>0.05) and (p = 0.072,0.498). The results of comparing average temperatures indicated no statistically significant difference between the outdoor air temperatures of Meymand (24.76 °C with std deviation of 2.73) and Shahr-e Babak (26.38 °C with std deviation of 2.14), with (p-value>0.05) and (df = 122). To compare R.H in these two locations, the Mann-Whitney *U* test was used due to the non-normality of the data [40] (p-value <0.05 and p = 0.00), demonstrating no significant difference in average R.H between Meymand and Shahr-e Babak ($\bar{Z}$ = -1.485, sig>0.05). Due to the geographical proximity and location within a similar climatic zone [30,31], a significant similarity was observed in the average environmental parameters of these two locations. Statistically, during data collection, no significant difference was observed between the temperature and R.H.

### 3.2 Evaluation of indoor environment building parameters

**Fig. 6:**
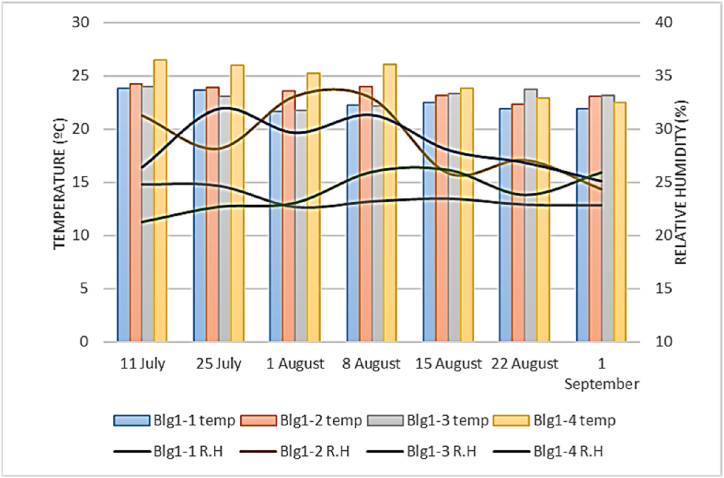
Indoor temperature and R.H in Meymand buildings.

**Fig. 7:**
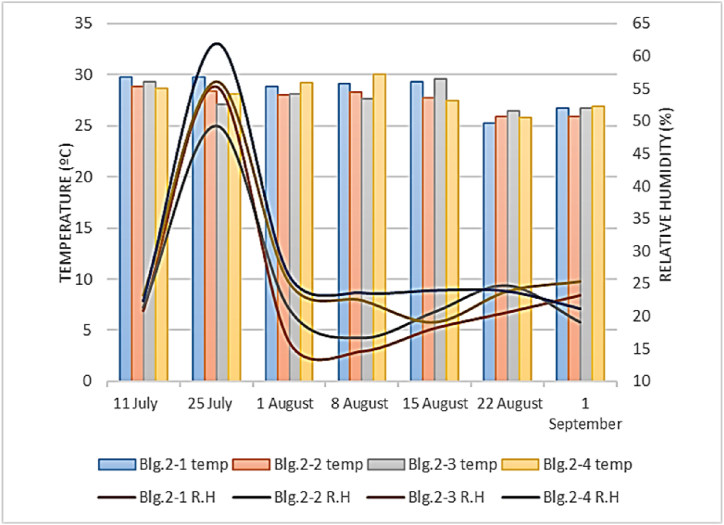
Indoor temperature and R.H in Shahre Babak buildings.

**Fig. 8:**
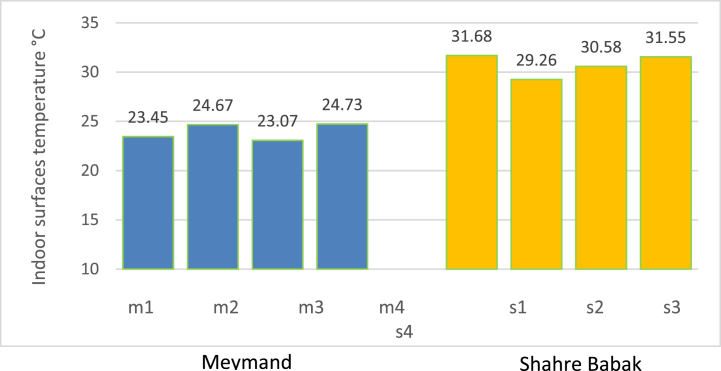
Surface temperature average comparison between Meymand buildings and Shahre Babak selected buildings.

Fig. 6, Fig. 7 show the temperature and R.H variation in four evaluated buildings in Meymand and Shahr-e Babak. The average indoor temperature in the four buildings of Meymand was 23.12 °C, with a std deviation of 0.40. The average indoor temperature in the four assessed buildings in Shahr-e Babak was 27.73 °C, with a std deviation of 0.89. For internal R.H. in these buildings in Meymand, the average was 25.55 %, with a std deviation of 0.85. For Shahr-e Babak buildings, this parameter was 27.43 % with std deviation of 8.04. The Kolmogorov-Smirnov test has been used to check the normality of the temperature data in both groups. The results indicated that the data were normally distributed (p-value>0.05) (sig = 0.414, 0.205). Therefore, an independent sample *t*-test used to compare their means, indicating a significant difference between the average indoor temperatures of the four Meymand buildings and four Shahr-e Babak buildings (sig = 0.259), (F = 1.30), (df = 54). Additionally, the normality of indoor R.H data in both groups was evaluated (p-value<0.05), (sig = 0.000, 0.001), showing that the distribution of indoor R.H data in both categories is not normal. Therefore, the non-parametric Mann-Whitney *U* test was employed to compare the average R.H between the two groups, revealing a significant difference between the average internal humidity of the four buildings in Meymand and Shahr-e Babak ($\bar{Z}$ = 1.222) (sig = 0.222). Measuring the temperature of the internal surfaces of the buildings was also done in both groups, and, at the time of data collection, the indoor surface temperature of walls, ceiling, floor, and windows was collected, and the overall average of these temperatures is shown in Fig. 8. The average temperature of indoor surfaces in Meymand buildings was recorded as 23.98 °C with a standard deviation of 2.86. At the same time, this average for the indoor surfaces of selected modern buildings in Shahre Babak was calculated as 30.76 °C with a standard deviation of 2.64. This is while the average difference between surface temperature and indoor temperature in Meymand's buildings was found to be 0.25 °C, but in modern buildings, this difference was calculated to be 3.03 °C. The small difference between the temperature of the surfaces and the indoor air temperature of Rock-cut buildings shows, that these buildings' indoor temperature is influenced by wall temperature and that the uniformity of the temperature of the stone wall surfaces of the building affects the uniformity and slow changes of the building's indoor temperature. However, in modern buildings, the large difference between the temperature of the surfaces and the indoor air temperature is caused by using the cooling system and energy consumption to create this temperature difference and control the level of thermal comfort. If the cooling system is not used, the indoor air temperature of these buildings can become hotter because of the temperature of the walls, and the thermal comfort level tends to be a warm sensation level.

### 3.3 Evaluation of outdoor PMV

**Fig. 9:**
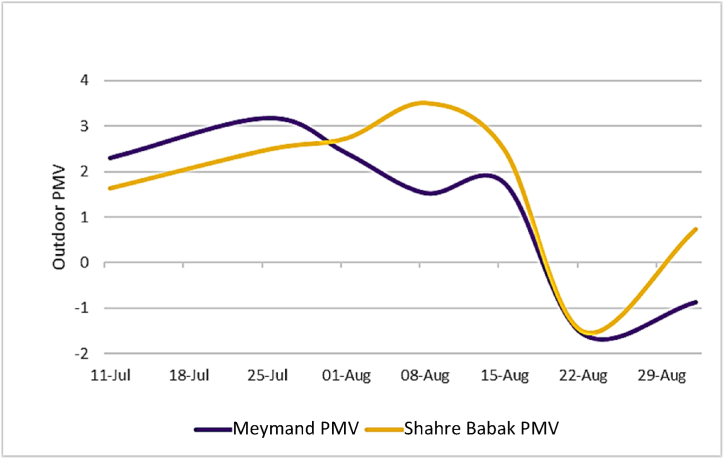
Outdoor PMV change in Meymand and Shahre Babak.

Table 5 and Fig. 9 illustrate the level and pattern of changes in outdoor PMV during the summer in Meymand and Shahr-e Babak. The daily average outdoor PMV in Meymand is 0.96, with a std deviation of 1.47, while this index in Shahr-e Babak is 1.52 with a std deviation of 0.57. The lowest average outdoor PMV during the day at 6 a.m. on data collection days was −0.314, with a std deviation of 2.192, moreover, the highest average outdoor PMV during the day at 3 p.m. in Meymand was recorded at 3.45, with a std deviation of 1.46. Simultaneously, this average for Shahr-e Babak at 6 a.m. was −0.7, with a std deviation of 1.71. At 3 p.m., the maximum PMV level was 3.81, with a std deviation of 1.76. The main reason for the significant variations in this index is the elevated temperature difference throughout the day, resulting from the low R.H level due to the placement of these two locations in a semi-arid climatic zone.

**Table 5:** Indoor and outdoor PMV monthly average in Meymand and Shahre Babak.

Month	Location	Indoor PMV	Outdoor PMV
July	Meymand	−0.32	2.73
August		−0.67	1.02
September		−0.86	−0.87
Average		−0.61	0.96
Standard Deviation		0.22	1.47
July	Shahre Babak	1.15	2.05
August		0.75	1.79
September		0.42	0.73
Average		0.77	1.52
Standard Deviation		0.29	0.57

### 3.4 Evaluation of indoor PMV

**Fig. 10:**
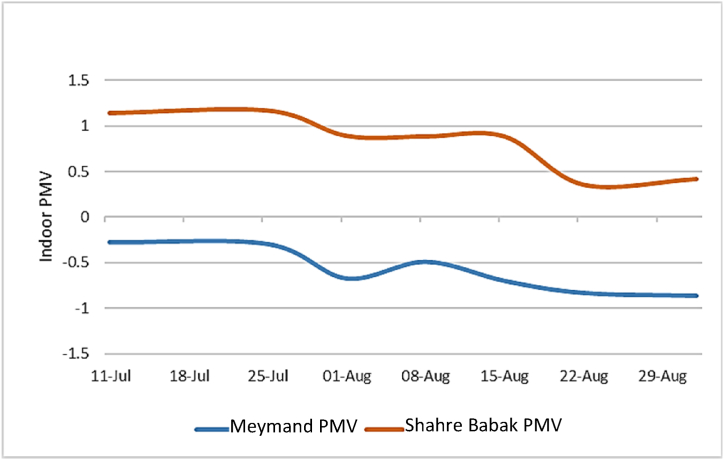
Indoor PMV change in Meymand and Shahre Babak buildings.

As shown in Table 5 and Fig. 10, the level of PMV variations in the indoor environment of the four selected buildings in Meymand and Shahr-e Babak during the study period indicates that the average indoor PMV for the buildings in Meymand is −0.61 with std deviation of 0.22, while it is 0.77 with std deviation of 0.29 in Shahr-e Babak. The average daily indoor PMV level for these buildings in Meymand is −0.612 with a std deviation of 0.233, while for Shahr-e Babak is 0.817 with a std deviation of 0.456. The normality test for the indoor daily PMV data showed that the distribution of these data is not normal (sig = 0.001). Therefore, a non-parametric Mann-Whitney *U* test has been used to compare the significance of the daily indoor PMV averages between the two groups, indicating no significant difference ($\bar{Z}$ = -5.982) (sig<0.05). The obtained average PMV values of both groups are close to the comfort level. Despite the proximity of PMV levels in the Meymand and Shahr-e Babak buildings and their placement within the desirable thermal comfort range, it is worth noting that the comfort level is achieved without energy consumption in the Meymand buildings. On the other hand, in the Shahr-e Babak buildings, comfort is approached during the day using a cooling system. Regarding comfort sensation, the PMV level in the Meymand buildings, without energy consumption, falls within the cool sensation PMV range of −0.612. However, despite the energy consumption for cooling in the Shahr-e Babak buildings, the PMV level is 0.817 with a warm sensation.

### 3.5 Correlation between outdoor temperature and PMV

Based on the Pearson correlation test, a statistically significant correlation (p-value<0.001) (r = 0.655) has been observed between outdoor temperature and PMV level inside the buildings during the day in Meymand. The same test was used for Shahr-e Babak buildings, revealing a significant correlation, as well (p-value <0.001) (r = 0.647). Notably, the correlation between outdoor temperature and indoor PMV in Meymand is lower than in Shahr-e Babak. The rate of temperature changes outside has a less pronounced effect on the indoor PMV in Meymand buildings than in Shahr-e Babak buildings. Remarkably, Meymand buildings did not utilize any cooling systems, while Shahr-e Babak buildings used a cooling system throughout the day. Despite the absence of energy consumption in Meymand buildings, the PMV is less responsive to outdoor temperature changes than the amount in Shahr-e Babak buildings.

## 4 Discussion

This research aimed to evaluate the thermal comfort level in the traditional architectural structures of the Meymand Cultural Heritage Complex in Meymand and compare it with modern buildings in Shahr-e Babak. Simultaneously, the study utilized the calculation of the thermal comfort index PMV during the summer of 2022. The evaluation of external climatic parameters in Meymand and Shahr-e Babak revealed that the average air temperature during the recorded days in the summer of 2022 was 24.76 °C in Meymand and 26.8 °C in Shahr-e Babak, showing no statistically significant difference in the daily outdoor temperature averages between the two locations (p-value >0.05) (df = 122). Additionally, the average outdoor R.H. for Meymand and Shahr-e Babak was 26.39 % and 28.45 %, respectively, without any significant difference (sig >0.05) ($\bar{Z}$ = -1.485). The proximity of the average climatic parameters in these two locations is attributed to their geographical closeness (less than 30 km) and their placement in a similar climatic zone. The primary difference is their elevation above the sea level in Meymand and Shahr-e Babak at 2200 and 1850 m, respectively.

At the same time, the indoor climatic parameters of temperature and R.H. have been recorded in four selected buildings in Meymand and Shahr-e Babak. The average indoor temperature for the four buildings in Meymand during summer days was 23.12 °C, while it was 27.73 °C for the four buildings in Shahr-e Babak, indicating a significant difference (sig = 0.259, sig>0.05). Additionally, the average indoor R.H. for these four buildings was 25.55 % and 27.43 % in Meymand and Shahr-e Babak, respectively, showing a significant difference (sig = 0.2. sig>0.05). The significant differences in environmental parameters between the groups of buildings stem from the fundamental architectural distinctions. The rock-cut architecture relies on the thermal mass of the earth and the substantial thickness of the stone layers in the settlement, along with the low heat exchange coefficient of the pyroclastic stones, which results in minimal temperature fluctuations inside these buildings, with the interior temperature being more influenced by the thermal properties of the stone context than the external temperature. In contrast, modern architecture, characterized by higher heat exchange coefficients in materials like brick, cement mortar, and concrete, coupled with larger window surfaces and weaker insulation, experiences more significant temperature variations. Further, the rate of temperature change inside these buildings is higher, which is more responsive to external temperature fluctuations.

During the day, the PMV index was calculated for the selected four buildings in Meymand and Shahr-e Babak. The results indicated that the PMV level in the buildings of Meymand sets within the semi-cool range of −0.612, while the PMV index during the day was within a warm sensation range in the selected four buildings in Shahr-e Babak by 0.817. It is worth noting that the indoor PMV level in the selected buildings in Shahr-e Babak during the day was achieved with the assistance of an air conditioning cooling system and energy consumption. In contrast, a cool comfort level was established without energy consumption in Meymand, which relies on the passive systems available in the buildings. Furthermore, the correlation test between the indoor PMV level and outside temperature for both groups showed that the buildings Meymand exhibited a lower correlation with external temperature changes compared to the buildings in Shahr-e Babak, which is attributed to the higher dependence of buildings in Meymand on the internal thermal stability resulting from the heat of the stone settlement and the lower heat exchange surface due to the thickness of its constituent layers.

In addition, the energy consumption of the cooling systems was collected in the four selected buildings in Shahr-e Babak. Each building used evaporative coolers during the day to reduce the PMV level and bring it closer to the comfort level. The average consumption of each device was 3078 kJ, resulting in an approximate 7.7 kW energy usage per residential unit daily between 9 a.m.; and 6 p.m. This energy consumption averaged 692 kW per building unit during the three-month summer period. Despite the high energy consumption, the daily PMV level in these buildings was 0.817. This falls within a warm sensation range, which can be attributed to inadequate and poor thermal insulation, improper orientation, thin layers of building materials, and a high surface area of openings in modern architecture. In contrast, the buildings in Meymand achieved an internal PMV level of −0.612 for the four buildings without using any energy, placing them in the cool sensation range.

The results from comparing the indoor thermal comfort of buildings in Meymand with Shahr-e Babak suggest that vernacular buildings are designed and constructed more sustainably and regionally adaptable. The finding aligns with that of other related studies [16]; by modeling and calculating indoor thermal comfort levels and assessing residents' comfort perceptions using questionnaires in traditional and modern residential buildings in Lebanon, it was found that traditional buildings offer higher comfort performance, which is attributed to their thicker building layers, appropriate height, and utilization of thermal mass. This finding is consistent with the rock-cut architectural features of Meymand buildings, and the presence of thick stone layers has provided a settlement to protect the indoor temperature of these buildings. However, the inadequate orientation of modern buildings is identified as the main reason for their high energy consumption and lower thermal comfort levels. A point that also applies to the selected modern buildings of the current research [17].demonstrated that approximately 88 % of residents reported feeling thermal comfort perception in traditional buildings in Ethiopia. In contrast, in modern buildings, only 22 % of residents reported comfort, and 83 % of inhabitants in traditional buildings believed that temperature changes in these structures occur very gradually. Furthermore, around 80 % considered these buildings well-adapted to climatic conditions which corresponds to the case of Meymand buildings with the calculated PMV of −0.612 in the range of neutral comfort feeling for its residents without energy consumption. In another study [19], modeled the thermal comfort and energy consumption in modern reinforced concrete buildings by focusing on traditional buildings in the warm and dry climate of Algeria. The findings indicated that modern concrete buildings lack suitable thermal perception in this climate. However, with the addition of proper shading and thermal insulation, the thermal comfort level increased by 35 %, and energy consumption decreased by 22.73 %. Carrying out these physical changes in the studied modern architectural buildings can also bring their thermal comfort to the neutral range without consuming energy [22]. conducted a study on the thermal comfort level of Tibetan houses in the Sichuan Plateau, China, and demonstrated that traditional buildings maintain a temperature range between 19 and 24 °C during the summer and exhibit better thermal comfort performance without energy consumption than other structures. The buildings constructed with stone showed better performance and could absorb approximately 19.9 % of solar energy. The temperature range calculated for Meymand buildings was also obtained as an average of 23.12, which is close to the findings of this research. In another study [18], compared the thermal comfort levels of buildings and residents' comfort perceptions in a rural and urban region in Wuhan, China by using questionnaires. The findings revealed that the residents in rural buildings have lower expectations of comfort compared to those in urban buildings during the winter. In contrast, the opposite was confirmed in the summer, which attributed to the better thermal comfort performance of rural buildings during the summer. The findings of this research and its comparison are consistent with the calculations made to determine the level of summer indoor thermal comfort in rural rock-cut buildings in Iran. In the same way [41], compared the thermal comfort of a historical market and a modern market in the semi-arid climate of Shiraz, Iran, during the summer by using a questionnaire. They found that traditional markets exhibit better thermal comfort performance than modern markets. The role of shading structures in traditional markets is crucial, with 73 % of users reporting discomfort in modern markets during the afternoon hours. A problem that is also consistent with the calculation of the thermal comfort level of modern buildings in the current research, is that in selected modern buildings, despite the use of cooling systems, the thermal comfort level is higher than the neutral range and includes a feeling of relatively warm comfort. Further [25,26], found that residents of historical neighborhoods in Isfahan experienced higher thermal comfort during the warm season in the semi-arid climate compared to the residents of contemporary neighborhoods. In the cold season, they reported higher comfort perception levels in the new neighborhoods, aligning with the calculated thermal comfort levels in both contexts. Similarly [42], compared the comfort range in the historical Fin Garden complex in Kashan with the historical fabric of the city in a warm and arid climate and found that the comfort range in this historical garden is 1.52 % higher than the comfort range in the historical fabric, despite the higher perceived comfort temperature range in this historical setting. As with [27], developed a building model and placed it in the climatic conditions of six different cities in various climatic zones of Iran according to ASHRAE standards. Without using an HVAC system and employing natural ventilation modeling, they found that the building is within the comfort range in 69 % of cases in a cold climate, and the lowest comfort level (12 %) was observed in a warm and humid climate. The thermal comfort performance of the building improved by 8–12 % by adding an insulation layer in the modeling. Which shows the role of thermal stability of building layers in creating comfortable conditions inside them. A problem that is less observed in modern buildings, but it exists intrinsically in rock-cut buildings due to the nature of their architectural stone layers and the thermal mass of the excavation settlement. In another study [43], evaluated the impact of indigenous materials and construction methods on reducing energy consumption in buildings in northern Iran, located in a moderately humid climate. The modeling revealed that the electricity consumption of three buildings with regional vernacular architecture is 176 kW per year less than similar buildings with modern architecture. The difference is attributed to the specific materials and structural systems used in these buildings. This amount of difference in energy consumption between traditional and modern buildings in northern Iran and the amount of near-zero summer consumption of rock-cut buildings for cooling shows the low level of energy consumption in vernacular buildings of Iran. Further [23], studied the summer thermal comfort of traditional buildings in eastern Iran, situated in a warm and arid climate, across six different villages by using questionnaires. They found that the passive natural ventilation system employed in these buildings can contribute to comfort during 51 % of hot days throughout the year. Considering that the rock-cut buildings of Iran are in the range of thermal comfort feeling close to neutral during the peak of heat, so, they maintain this condition for a large part of the days of the year and have a level of comfort for living.

By comparing the results of similar studies with those of the present study, it can be concluded that vernacular architectural structures exhibit suitable thermal comfort performance during warm seasons. Overall, they require less energy for cooling compared to modern buildings. The increased utilization of passive energy systems in vernacular architecture can contribute to this reduced energy consumption. Furthermore, the thermal comfort index of traditional buildings is closely consistent with the satisfaction level of their residents, particularly in warm seasons, surpassing that of residents in modern architectural structures. Therefore, vernacular architecture can provide higher environmental sustainability without energy consumption or by utilizing energy from renewable sources, which aligns with concept of the closer alignment of vernacular architecture, especially vernacular architecture, with environmental and energy sustainability. Despite the geographical similarities between the two places and their close distance, in one case, large amounts of energy were used to create thermal comfort, but, at the same time, in another case, without energy consumption, the buildings are even in a more suitable temperature comfort condition. The difference in the essence of architecture and the materials used in these two places is the main cause of this difference in energy consumption and thermal comfort level. It seems that the level of thermal comfort depends more on the architectural conditions of the buildings such as design features, materials used, and the building structures along with the effect of microclimatic factors. Phenomena like height above sea level and geographical conditions have less impact on thermal comfort than architectural features. The materials used and their characteristics, such as the thermal phase of the materials, the use of thermal insulation, and the thickness of the layers of materials, are the determining factors in the internal temperature stability of the building. Digging in low heat exchange coefficients pyroclastic rock mass and the high average thickness stone layers helps Meymand rock-cut architecture buildings to create these conditions. On the other hand, the design features of the building, such as the small area of doors and windows, along with the depth of the building's penetration into the rock mass of the excavation context, create an insulating environment by locking the human living space in thick stone walls. The total of these conditions has led to space creation which has the lowest temperature exchange with the outdoor environment and the temperature of the earth's surface and the temperature stability of the mass rock in which it is excavated affects its indoor temperature. In addition, the low thickness of building layers in modern architecture, the use of materials with an incommensurate thermal phase, the lack of thermal insulation, the large contact surface of the building with sunlight and open air, and the large area of glass doors and windows, are other parameters which provide the possibility of temperature exchange between indoor and outdoor air conditions. The result of these conditions is the need for energy consumption to control the thermal comfort inside the building.

The main limitation of the results in this study lies in the restricted generalizability to all types of vernacular architecture. This study focused solely on a subset of buildings within the vernacular architecture in a semi-arid climate, necessitating further studies to understand the thermal perception and energy consumption of other examples of vernacular architecture in various climates. More similar research on vernacular architecture can lead to the recognition of design and architectural features, the choice of materials and how to use them, and the structural conditions of the building which have a fundamental role in creating thermal comfort in native buildings without energy consumption and their patterns can be used in the form of redesign in climate optimization and reducing energy consumption of modern architectural buildings. Despite the limitation, our knowledge of the nature and behavior of vernacular architecture has expanded, facilitating its adaptation to contemporary life and inspiring contemporary designs within this architectural style. Additionally, this study has highlighted new values associated with vernacular architecture, which should be considered in the preservation and restoration processes of this type of vernacular architecture. This means that the protection of the bio-climatic characteristics of vernacular architectural examples should be given more attention in their maintenance and restoration plans due to the existence of architectural sustainability values and a model for today's design. Conducting more research related to the modeling of the thermal perception of vernacular architecture, analyzing the influence of environmental and architectural factors on the thermal comfort of these buildings, and assessing the sustainability of different subtypes of this architecture in the future can contribute to a broader understanding. This knowledge can be patterned for application in contemporary architecture, which can enhance our understanding of the multifaceted aspects of vernacular architecture for future use.

## 5 Conclusion

In this study, a comparison is done between the daily internal thermal comfort index (PMV) levels of four rock-cut buildings in the UNESCO World Heritage Site of Meymand and four modern buildings in Shahr-e Babak by using a field study approach and quantitative evaluation of their climatic data. The data included the outdoor and indoor temperatures of the buildings, air flow rates, and R.H. levels. The study was conducted during the summer of 2022, which experienced the highest temperatures in this geographical and climatic region. Using the data obtained from the field study, the indoor PMV levels were calculated for both groups. In the Meymand buildings, the average PMV was approximately determined −0.612, indicating a range of semi-cool thermal comfort. As for the Shahr-e Babak buildings, the PMV was found to be 0.817, placing it in the range of warm thermal comfort. The comparison of these two averages revealed a significant difference in thermal comfort levels between traditional rock-cut and modern buildings during the summer season, with rock-cut buildings exhibiting more favorable thermal perceptions. A higher correlation was observed between the external temperature and internal PMV in modern buildings compared to rock-cut buildings. The difference is attributed to the rock-cut buildings' dependence on the ground temperature. During the data collection period, the energy consumption to bring the rock-cut buildings within the comfort range was zero on scorching summer days. The buildings achieved semi-cool thermal comfort without energy consumption. In contrast, modern buildings, on average, consumed about 7.7 kW per residential unit solely to approach the comfort range. Despite the high energy consumption, the PMV levels in these modern buildings were within the warm range.

In general, rock-cut buildings in Meymand exhibit a more favorable thermal comfort performance compared to modern buildings during the summer season, which can be related to some factors such as the substantial thickness of the building layers, thermal phase of the rock foundation of the excavated structures, low heat exchange coefficient, minimal surface area of openings, proper orientation, and use of natural ventilation systems.

The importance of saving energy and reducing its consumption is a model of vernacular architecture that is always part of the essence of this architecture and has been neglected in its architectural studies. This research has pointed to the new recognition of an exceptional type of vernacular architecture which shows that it is possible to plan and design a sustainable architecture type with minimal facilities and consumption of resources and materials that can provide human living conditions without energy consumption. This research is a nudge to today's architects to pay more attention to the climatic issues of native architecture and use its patterns after recognizing them in their designs. It is possible to provide the conditions of human life with the least consumption of resources and energy with the facilities of vernacular architecture. This significant matter is more important in a country like Iran, which has paid less attention to energy conservation in architecture.

The results indicated that traditional rock-cut buildings can create thermal comfort without energy consumption during the warm season. The architectural strategies employed in these buildings for achieving thermal comfort could serve as a model for incorporation into modern architecture. On the other hand, the lack of adherence to climatic design principles, low construction quality, and minimal emphasis on energy efficiency in modern urban architecture has led to the construction of buildings that require high energy consumption to establish a thermal comfort range during the summer season in warm and dry or semi-warm and dry climates. This comparison underscores the importance of further studying vernacular rock-cut buildings in sustainability, energy consumption, and architectural conservation.

## Data availability

Data is available via the corresponding authors upon reasonable requests for academic purposes.

## CRediT authorship contribution statement

**Mohammad Mangeli:** Writing – review & editing, Writing – original draft, Data curation, Conceptualization. **Farshid Aram:** Visualization, Project administration, Methodology. **Reza Abouei:** Validation, Software, Formal analysis.

## Declaration of competing interest

The authors declare that they have no known competing financial interests or personal relationships that could have appeared to influence the work reported in this paper.
