# Supplementary material for: Energy consumption and thermal comfort of rock-cut and modern buildings
Source: Heliyon. 2024 Jul 8;10(14):e34217. doi: 10.1016/j.heliyon.2024.e34217 (PMC11295850; doi:10.1016/j.heliyon.2024.e34217)
Supplement: Multimedia component 1 [file mmc1.docx]

RayMan 1.2 © 2000

Meteorological Institute, University of Freiburg, Germany

place: Meymand

Horizon limitation: 0.0% sky view factor: 1.000

geogr. longitude: 55°22' latitude: 30°13' timezone: UTC +3.3 h

personal data: height: 1.72 m weight: 71.4 kg age: 42 a sex: m clothing: 0.5 clo activity: 60.0 W

day of time sun sun Gact Sact Dact Ts Ta Tmrt PMV PET SET*

date year h:mm rise set W/m2 W/m2 W/m2 °C °C °C °C °C

11.7. 23 192 3:00 4:47 18:36 0 0 0 16.8 19.5 7.3 -4.1 12.9 1.5

day of time sun sun Gact Sact Dact Ts Ta Tmrt PMV PET SET*

date year h:mm rise set W/m2 W/m2 W/m2 °C °C °C °C °C

11.7. 23192 6:00 4:47 18:36 106 41 64 19.5 20.0 19.5 -2.7 17.0 9.5

day of time sun sun Gact Sact Dact Ts Ta Tmrt PMV PET SET*

date year h:mm rise set W/m2 W/m2 W/m2 °C °C °C °C °C

11.7. 23 192 9:00 4:47 18:36 706 546 160 32.5 24.0 49.2 -0.1 25.5 20.8

day of time sun sun Gact Sact Dact Ts Ta Tmrt PMV PET SET*

date year h:mm rise set W/m2 W/m2 W/m2 °C °C °C °C °C

11.7. 23192 12:00 4:47 18:36 963 815 149 37.2 29.0 48.9 1.4 29.1 23.5

day of time sun sun Gact Sact Dact Ts Ta Tmrt PMV PET SET*

date year h:mm rise set W/m2 W/m2 W/m2 °C °C °C °C °C

11.7. 23 192 15:00 4:47 18:36 706 546 160 42.2 35.0 57.5 4.6 42.7 33.9

day of time sun sun Gact Sact Dact Ts Ta Tmrt PMV PET SET*

date year h:mm rise set W/m2 W/m2 W/m2 °C °C °C °C °C

11.7. 23 192 18:00 4:47 18:36 106 41 64 35.5 35.8 35.5 3.3 35.9 27.9

day of time sun sun Gact Sact Dact Ts Ta Tmrt PMV PET SET*

date year h:mm rise set W/m2 W/m2 W/m2 °C °C °C °C °C

11.7. 23 192 21:00 4:47 18:36 0 0 0 27.8 30.0 19.5 0.1 24.3 17.6

day of time sun sun Gact Sact Dact Ts Ta Tmrt PMV PET SET*

date year h:mm rise set W/m2 W/m2 W/m2 °C °C °C °C °C

11.7. 23 192 0:00 4:47 18:36 0 0 0 29.7 31.0 20.6 0.5 25.2 17.9

---------------------------------------------------------------------------------------------------------------------------------------------------

day of time sun sun Gact Sact Dact Ts Ta Tmrt PMV PET SET*

date year h:mm rise set W/m2 W/m2 W/m2 °C °C °C °C °C

25.7. 23 206 3:00 4:55 18:31 0 0 0 18.7 20.0 11.9 -3.8 14.3 3.3

day of time sun sun Gact Sact Dact Ts Ta Tmrt PMV PET SET*

date year h:mm rise set W/m2 W/m2 W/m2 °C °C °C °C °C

25.7. 23 206 6:00 4:55 18:31 88 34 53 20.9 21.0 21.9 -2.8 17.1 8.3

day of time sun sun Gact Sact Dact Ts Ta Tmrt PMV PET SET*

date year h:mm rise set W/m2 W/m2 W/m2 °C °C °C °C °C

25.7. 23 206 9:00 4:55 18:31 696 538 158 43.0 27.6 56.0 3.3 38.6 33.5

day of time sun sun Gact Sact Dact Ts Ta Tmrt PMV PET SET*

date year h:mm rise set W/m2 W/m2 W/m2 °C °C °C °C °C

25.7. 23 206 12:00 4:55 18:31 959 813 146 44.4 29.5 53.7 2.8 35.9 30.2

day of time sun sun Gact Sact Dact Ts Ta Tmrt PMV PET SET*

date year h:mm rise set W/m2 W/m2 W/m2 °C °C °C °C °C

25.7. 23 206 15:00 4:55 18:31 696 538 158 45.2 33.0 58.7 4.7 43.3 36.5

day of time sun sun Gact Sact Dact Ts Ta Tmrt PMV PET SET*

date year h:mm rise set W/m2 W/m2 W/m2 °C °C °C °C °C

25.7. 23 206 18:00 4:55 18:31 88 34 53 31.2 31.6 31.3 1.9 30.9 25.7

day of time sun sun Gact Sact Dact Ts Ta Tmrt PMV PET SET*

date year h:mm rise set W/m2 W/m2 W/m2 °C °C °C °C °C

25.7. 23 206 21:00 4:55 18:31 0 0 0 27.3 28.6 20.5 0.0 23.6 18.2

day of time sun sun Gact Sact Dact Ts Ta Tmrt PMV PET SET*

date year h:mm rise set W/m2 W/m2 W/m2 °C °C °C °C °C

25.7. 23 206 0:00 4:55 18:31 0 0 0 25.2 27.0 18.7 -0.6 22.1 16.4

__________________________________________________________________________________________

day of time sun sun Gact Sact Dact Ts Ta Tmrt PMV PET SET*

date year h:mm rise set W/m2 W/m2 W/m2 °C °C °C °C °C

1.8. 23 213 3:00 4:59 18:26 0 0 0 17.4 19.5 8.7 -4.1 13.1 1.0

day of time sun sun Gact Sact Dact Ts Ta Tmrt PMV PET SET*

date year h:mm rise set W/m2 W/m2 W/m2 °C °C °C °C °C

1.8. 23 213 6:00 4:59 18:26 75 29 47 16.3 17.1 14.4 -4.5 12.5 0.9

day of time sun sun Gact Sact Dact Ts Ta Tmrt PMV PET SET*

date year h:mm rise set W/m2 W/m2 W/m2 °C °C °C °C °C

1.8. 23 213 9:00 4:59 18:26 689 533 157 35.8 21.0 49.1 0.3 28.3 24.1

day of time sun sun Gact Sact Dact Ts Ta Tmrt PMV PET SET*

date year h:mm rise set W/m2 W/m2 W/m2 °C °C °C °C °C

1.8. 23 213 12:00 4:59 18:26 955 810 145 39.9 28.0 50.3 1.5 30.6 24.9

day of time sun sun Gact Sact Dact Ts Ta Tmrt PMV PET SET*

date year h:mm rise set W/m2 W/m2 W/m2 °C °C °C °C °C

1.8. 23 213 15:00 4:59 18:26 689 533 157 44.8 35.5 58.7 5.1 44.8 36.2

day of time sun sun Gact Sact Dact Ts Ta Tmrt PMV PET SET*

date year h:mm rise set W/m2 W/m2 W/m2 °C °C °C °C °C

1.8. 23 213 18:00 4:59 18:26 75 29 47 34.2 35.0 32.3 2.7 33.7 26.5

day of time sun sun Gact Sact Dact Ts Ta Tmrt PMV PET SET*

date year h:mm rise set W/m2 W/m2 W/m2 °C °C °C °C °C

1.8. 23 213 21:00 4:59 18:26 0 0 0 29.1 31.3 21.2 0.6 25.8 19.6

day of time sun sun Gact Sact Dact Ts Ta Tmrt PMV PET SET*

date year h:mm rise set W/m2 W/m2 W/m2 °C °C °C °C °C

1.8. 23 213 0:00 4:59 18:26 0 0 0 26.8 28.2 18.3 -0.7 22.4 13.9

__________________________________________________________________________________________

day of time sun sun Gact Sact Dact Ts Ta Tmrt PMV PET SET*

date year h:mm rise set W/m2 W/m2 W/m2 °C °C °C °C °C

8.8. 23 220 3:00 5:03 18:20 0 0 0 18.4 20.0 8.8 -4.3 13.4 -0.5

day of time sun sun Gact Sact Dact Ts Ta Tmrt PMV PET SET*

date year h:mm rise set W/m2 W/m2 W/m2 °C °C °C °C °C

8.8. 23 220 6:00 5:03 18:20 62 23 39 15.9 17.0 11.5 -5.1 11.3 -2.8

day of time sun sun Gact Sact Dact Ts Ta Tmrt PMV PET SET*

date year h:mm rise set W/m2 W/m2 W/m2 °C °C °C °C °C

8.8. 23 220 9:00 5:03 18:20 680 525 156 32.4 22.8 47.7 -0.4 25.1 20.5

day of time sun sun Gact Sact Dact Ts Ta Tmrt PMV PET SET*

date year h:mm rise set W/m2 W/m2 W/m2 °C °C °C °C °C

8.8. 23 220 12:00 5:03 18:20 949 805 144 40.0 28.5 49.7 1.5 31.0 24.9

day of time sun sun Gact Sact Dact Ts Ta Tmrt PMV PET SET*

date year h:mm rise set W/m2 W/m2 W/m2 °C °C °C °C °C

8.8. 23 220 15:00 5:03 18:20 680 525 156 38.5 33.0 54.0 3.3 37.5 29.4

day of time sun sun Gact Sact Dact Ts Ta Tmrt PMV PET SET*

date year h:mm rise set W/m2 W/m2 W/m2 °C °C °C °C °C

8.8. 23 220 18:00 5:03 18:20 62 23 39 32.3 33.0 27.6 1.7 29.0 21.8

day of time sun sun Gact Sact Dact Ts Ta Tmrt PMV PET SET*

date year h:mm rise set W/m2 W/m2 W/m2 °C °C °C °C °C

8.8.23 220 21:00 5:03 18:20 0 0 0 27.8 29.0 18.3 -0.4 23.0 14.0

day of time sun sun Gact Sact Dact Ts Ta Tmrt PMV PET SET*

date year h:mm rise set W/m2 W/m2 W/m2 °C °C °C °C °C

8.8. 23 220 0:00 5:03 18:20 0 0 0 22.6 24.0 13.1 -2.7 17.6 5.8

__________________________________________________________________________________________

day of time sun sun Gact Sact Dact Ts Ta Tmrt PMV PET SET*

date year h:mm rise set W/m2 W/m2 W/m2 °C °C °C °C °C

15.8. 23 227 3:00 5:08 18:13 0 0 0 22.5 23.8 11.9 -2.9 17.2 4.2

day of time sun sun Gact Sact Dact Ts Ta Tmrt PMV PET SET*

date year h:mm rise set W/m2 W/m2 W/m2 °C °C °C °C °C

15.8. 23 227 6:00 5:08 18:13 47 17 30 21.5 22.3 15.3 -3.4 16.2 2.5

day of time sun sun Gact Sact Dact Ts Ta Tmrt PMV PET SET*

date year h:mm rise set W/m2 W/m2 W/m2 °C °C °C °C °C

15.8. 23 227 9:00 5:08 18:13 669 514 155 31.2 26.4 48.1 0.1 25.2 19.7

day of time sun sun Gact Sact Dact Ts Ta Tmrt PMV PET SET*

date year h:mm rise set W/m2 W/m2 W/m2 °C °C °C °C °C

15.8. 23 227 12:00 5:08 18:13 941 798 143 41.9 30.0 51.2 2.3 33.9 27.2

day of time sun sun Gact Sact Dact Ts Ta Tmrt PMV PET SET*

date year h:mm rise set W/m2 W/m2 W/m2 °C °C °C °C °C

15.8. 23 227 15:00 5:08 18:13 669 514 155 37.6 33.2 53.2 3.3 36.9 28.6

day of time sun sun Gact Sact Dact Ts Ta Tmrt PMV PET SET*

date year h:mm rise set W/m2 W/m2 W/m2 °C °C °C °C °C

15.8. 23 227 18:00 5:08 18:13 47 17 30 31.6 32.5 25.4 1.3 27.5 20.6

day of time sun sun Gact Sact Dact Ts Ta Tmrt PMV PET SET*

date year h:mm rise set W/m2 W/m2 W/m2 °C °C °C °C °C

15.8. 23 227 21:00 5:08 18:13 0 0 0 28.4 30.0 19.5 0.0 24.2 16.5

day of time sun sun Gact Sact Dact Ts Ta Tmrt PMV PET SET*

date year h:mm rise set W/m2 W/m2 W/m2 °C °C °C °C °C

15.8. 23 227 0:00 5:08 18:13 0 0 0 26.4 28.0 17.4 -0.8 22.0 13.3

---------------------------------------------------------------------------------------------------------------------------------------------------

day of time sun sun Gact Sact Dact Ts Ta Tmrt PMV PET SET*

date year h:mm rise set W/m2 W/m2 W/m2 °C °C °C °C °C

23.8. 23 235 3:00 5:13 18:05 0 0 0 10.9 12.9 -0.5 -7.3 5.6 -13.1

day of time sun sun Gact Sact Dact Ts Ta Tmrt PMV PET SET*

date year h:mm rise set W/m2 W/m2 W/m2 °C °C °C °C °C

23.8. 23 235 6:00 5:13 18:05 30 12 19 10.6 12.2 2.1 -7.4 5.4 -13.0

day of time sun sun Gact Sact Dact Ts Ta Tmrt PMV PET SET*

date year h:mm rise set W/m2 W/m2 W/m2 °C °C °C °C °C

23.8. 23 235 9:00 5:13 18:05 655 505 150 21.9 16.0 39.5 -4.5 14.7 3.4

day of time sun sun Gact Sact Dact Ts Ta Tmrt PMV PET SET*

date year h:mm rise set W/m2 W/m2 W/m2 °C °C °C °C °C

23.8. 23 235 12:00 5:13 18:05 931 793 139 29.5 22.0 43.1 -2.1 20.3 11.8

day of time sun sun Gact Sact Dact Ts Ta Tmrt PMV PET SET*

date year h:mm rise set W/m2 W/m2 W/m2 °C °C °C °C °C

23.8. 23 235 15:00 5:13 18:05 655 505 150 33.7 28.5 50.0 1.2 28.6 23.1

day of time sun sun Gact Sact Dact Ts Ta Tmrt PMV PET SET*

date year h:mm rise set W/m2 W/m2 W/m2 °C °C °C °C °C

23.8. 23 235 18:00 5:13 18:05 30 12 19 26.1 27.7 18.7 -0.9 22.0 13.5

day of time sun sun Gact Sact Dact Ts Ta Tmrt PMV PET SET*

date year h:mm rise set W/m2 W/m2 W/m2 °C °C °C °C °C

23.8. 23 235 21:00 5:13 18:05 0 0 0 22.9 25.1 12.9 -2.1 18.6 8.2

day of time sun sun Gact Sact Dact Ts Ta Tmrt PMV PET SET*

date year h:mm rise set W/m2 W/m2 W/m2 °C °C °C °C °C

23.8. 23 235 0:00 5:13 18:05 0 0 0 20.3 23.3 10.2 -2.7 16.5 6.7

__________________________________________________________________________________________

day of time sun sun Gact Sact Dact Ts Ta Tmrt PMV PET SET*

date year h:mm rise set W/m2 W/m2 W/m2 °C °C °C °C °C

1.9. 23 244 3:00 5:18 17:54 0 0 0 11.9 14.4 1.8 -6.2 7.4 -7.7

day of time sun sun Gact Sact Dact Ts Ta Tmrt PMV PET SET*

date year h:mm rise set W/m2 W/m2 W/m2 °C °C °C °C °C

1.9. 23 244 6:00 5:18 17:54 13 6 7 11.1 14.0 2.5 -5.7 7.5 -4.5

day of time sun sun Gact Sact Dact Ts Ta Tmrt PMV PET SET*

date year h:mm rise set W/m2 W/m2 W/m2 °C °C °C °C °C

1.9. 23 244 9:00 5:18 17:54 636 490 146 25.5 17.1 40.8 -3.1 18.1 10.0

day of time sun sun Gact Sact Dact Ts Ta Tmrt PMV PET SET*

date year h:mm rise set W/m2 W/m2 W/m2 °C °C °C °C °C

1.9. 23 244 12:00 5:18 17:54 916 782 134 32.8 24.5 46.0 -0.7 23.6 17.4

day of time sun sun Gact Sact Dact Ts Ta Tmrt PMV PET SET*

date year h:mm rise set W/m2 W/m2 W/m2 °C °C °C °C °C

1.9. 23 244 15:00 5:18 17:54 636 490 146 35.6 30.0 50.6 2.0 32.0 25.5

day of time sun sun Gact Sact Dact Ts Ta Tmrt PMV PET SET*

date year h:mm rise set W/m2 W/m2 W/m2 °C °C °C °C °C

1.9. 23 244 18:00 5:18 17:54 13 6 7 24.7 26.0 16.2 -1.7 20.0 9.6

day of time sun sun Gact Sact Dact Ts Ta Tmrt PMV PET SET*

date year h:mm rise set W/m2 W/m2 W/m2 °C °C °C °C °C

1.9. 23 244 21:00 5:18 17:54 0 0 0 19.9 22.0 10.1 -3.4 15.4 3.7

day of time sun sun Gact Sact Dact Ts Ta Tmrt PMV PET SET*

date year h:mm rise set W/m2 W/m2 W/m2 °C °C °C °C °C

1.9. 23 244 0:00 5:18 17:54 0 0 0 0.1 2.8 -13.1 -11.0 -5.2 -28.7

RayMan 1.2 © 2000

Meteorological Institute, University of Freiburg, Germany

place: Shahre Babak

Horizon limitation: 0.0% sky view factor: 1.000

geogr. longitude: 55°12' latitude: 30°11' timezone: UTC +3.3 h

personal data: height: 1.72 m weight: 71.4 kg age: 42 a sex: m clothing: 0.5 clo activity: 60.0 W

day of time sun sun Gact Sact Dact Ts Ta Tmrt PMV PET SET*

date year h:mm rise set W/m2 W/m2 W/m2 °C °C °C °C °C

11.7. 22 192 3:00 4:48 18:37 0 0 0 20.1 20.9 12.3 -4.0 14.6 0.6

day of time sun sun Gact Sact Dact Ts Ta Tmrt PMV PET SET*

date year h:mm rise set W/m2 W/m2 W/m2 °C °C °C °C °C

11.7. 22 192 6:00 4:48 18:37 102 38 64 20.1 20.0 22.3 -3.7 15.4 3.9

day of time sun sun Gact Sact Dact Ts Ta Tmrt PMV PET SET*

date year h:mm rise set W/m2 W/m2 W/m2 °C °C °C °C °C

11.7. 22 192 9:00 4:48 18:37 700 530 170 27.8 22.0 47.8 -1.6 21.4 14.7

day of time sun sun Gact Sact Dact Ts Ta Tmrt PMV PET SET*

date year h:mm rise set W/m2 W/m2 W/m2 °C °C °C °C °C

11.7. 22 192 12:00 4:48 18:37 957 796 161 37.1 28.5 48.7 1.1 28.5 22.9

day of time sun sun Gact Sact Dact Ts Ta Tmrt PMV PET SET*

date year h:mm rise set W/m2 W/m2 W/m2 °C °C °C °C °C

11.7. 22 192 15:00 4:48 18:37 700 530 170 40.7 33.0 57.0 3.9 40.0 32.2

day of time sun sun Gact Sact Dact Ts Ta Tmrt PMV PET SET*

date year h:mm rise set W/m2 W/m2 W/m2 °C °C °C °C °C

11.7. 22 192 18:00 4:48 18:37 102 38 64 35.1 35.5 35.0 3.1 35.3 27.6

day of time sun sun Gact Sact Dact Ts Ta Tmrt PMV PET SET*

date year h:mm rise set W/m2 W/m2 W/m2 °C °C °C °C °C

11.7. 22 192 21:00 4:48 18:37 0 0 0 30.7 32.0 23.4 1.2 26.9 20.9

day of time sun sun Gact Sact Dact Ts Ta Tmrt PMV PET SET*

date year h:mm rise set W/m2 W/m2 W/m2 °C °C °C °C °C

11.7. 22 192 0:00 4:48 18:37 0 0 0 29.8 31.0 22.5 0.8 25.7 19.6

------------------------------------------------------------------------------------------------------------------------------------------------

day of time sun sun Gact Sact Dact Ts Ta Tmrt PMV PET SET*

date year h:mm rise set W/m2 W/m2 W/m2 °C °C °C °C °C

25.7. 23 206 3:00 4:56 18:31 0 0 0 22.7 24.0 15.5 -2.3 18.3 8.4

day of time sun sun Gact Sact Dact Ts Ta Tmrt PMV PET SET*

date year h:mm rise set W/m2 W/m2 W/m2 °C °C °C °C °C

25.7. 23 206 6:00 4:56 18:31 84 31 53 20.5 20.7 21.2 -2.8 17.0 8.3

day of time sun sun Gact Sact Dact Ts Ta Tmrt PMV PET SET*

date year h:mm rise set W/m2 W/m2 W/m2 °C °C °C °C °C

25.7. 23 206 9:00 4:56 18:31 691 523 168 33.4 22.5 51.0 0.2 26.7 23.2

day of time sun sun Gact Sact Dact Ts Ta Tmrt PMV PET SET*

date year h:mm rise set W/m2 W/m2 W/m2 °C °C °C °C °C

25.7. 23 206 12:00 4:56 18:31 953 794 158 41.4 24.3 51.7 1.2 30.3 26.3

day of time sun sun Gact Sact Dact Ts Ta Tmrt PMV PET SET*

date year h:mm rise set W/m2 W/m2 W/m2 °C °C °C °C °C

25.7. 23 206 15:00 4:56 18:31 691 523 168 44.2 31.8 58.5 4.6 42.4 36.7

day of time sun sun Gact Sact Dact Ts Ta Tmrt PMV PET SET*

date year h:mm rise set W/m2 W/m2 W/m2 °C °C °C °C °C

25.7. 23 206 18:00 4:56 18:31 84 31 53 35.2 35.6 34.9 3.9 36.3 31.5

day of time sun sun Gact Sact Dact Ts Ta Tmrt PMV PET SET*

date year h:mm rise set W/m2 W/m2 W/m2 °C °C °C °C °C

25.7.20 206 21:00 4:56 18:31 0 0 0 36.8 38.7 30.1 4.7 36.9 37.9

day of time sun sun Gact Sact Dact Ts Ta Tmrt PMV PET SET*

date year h:mm rise set W/m2 W/m2 W/m2 °C °C °C °C °C

25.7. 23 206 0:00 4:56 18:31 0 0 0 31.0 33.0 24.5 2.0 29.8 25.0

__________________________________________________________________________________________

day of time sun sun Gact Sact Dact Ts Ta Tmrt PMV PET SET*

date year h:mm rise set W/m2 W/m2 W/m2 °C °C °C °C °C

1.8. 23 213 3:00 5:00 18:27 0 0 0 23.8 26.0 15.4 -1.5 20.0 11.3

day of time sun sun Gact Sact Dact Ts Ta Tmrt PMV PET SET*

date year h:mm rise set W/m2 W/m2 W/m2 °C °C °C °C °C

1.8. 23 213 6:00 5:00 18:27 72 26 46 24.1 25.5 21.5 -1.1 21.5 14.8

day of time sun sun Gact Sact Dact Ts Ta Tmrt PMV PET SET*

date year h:mm rise set W/m2 W/m2 W/m2 °C °C °C °C °C

1.8. 23 213 9:00 5:00 18:27 684 517 166 35.8 26.0 51.4 1.1 30.1 24.7

day of time sun sun Gact Sact Dact Ts Ta Tmrt PMV PET SET*

date year h:mm rise set W/m2 W/m2 W/m2 °C °C °C °C °C

1.8. 23 213 12:00 5:00 18:27 949 792 157 45.1 33.0 54.1 3.6 39.1 31.2

day of time sun sun Gact Sact Dact Ts Ta Tmrt PMV PET SET*

date year h:mm rise set W/m2 W/m2 W/m2 °C °C °C °C °C

1.8. 23 213 15:00 5:00 18:27 684 517 166 38.6 35.0 54.4 4.0 39.9 30.2

day of time sun sun Gact Sact Dact Ts Ta Tmrt PMV PET SET*

date year h:mm rise set W/m2 W/m2 W/m2 °C °C °C °C °C

1.8. 23 213 18:00 5:00 18:27 72 26 46 33.5 34.0 28.8 2.2 30.8 22.7

day of time sun sun Gact Sact Dact Ts Ta Tmrt PMV PET SET*

date year h:mm rise set W/m2 W/m2 W/m2 °C °C °C °C °C

1.8. 23 213 21:00 5:00 18:27 0 0 0 29.5 31.0 19.0 0.4 24.9 17.1

day of time sun sun Gact Sact Dact Ts Ta Tmrt PMV PET SET*

date year h:mm rise set W/m2 W/m2 W/m2 °C °C °C °C °C

1.8. 23 213 0:00 5:00 18:27 0 0 0 27.0 28.0 16.3 -1.0 21.7 11.3

__________________________________________________________________________________________

day of time sun sun Gact Sact Dact Ts Ta Tmrt PMV PET SET*

date year h:mm rise set W/m2 W/m2 W/m2 °C °C °C °C °C

8.8. 23 220 3:00 5:04 18:21 0 0 0 19.6 21.0 9.6 -4.0 14.3 0.3

day of time sun sun Gact Sact Dact Ts Ta Tmrt PMV PET SET*

date year h:mm rise set W/m2 W/m2 W/m2 °C °C °C °C °C

8.8. 23 220 6:00 5:04 18:21 59 21 38 18.9 19.5 16.2 -4.1 14.2 1.6

day of time sun sun Gact Sact Dact Ts Ta Tmrt PMV PET SET*

date year h:mm rise set W/m2 W/m2 W/m2 °C °C °C °C °C

8.8. 23 220 9:00 5:04 18:21 675 509 165 34.7 25.0 50.8 0.7 28.7 23.8

day of time sun sun Gact Sact Dact Ts Ta Tmrt PMV PET SET*

date year h:mm rise set W/m2 W/m2 W/m2 °C °C °C °C °C

8.8. 23 220 12:00 5:04 18:21 943 787 156 44.7 35.0 56.4 4.5 42.2 33.3

day of time sun sun Gact Sact Dact Ts Ta Tmrt PMV PET SET*

date year h:mm rise set W/m2 W/m2 W/m2 °C °C °C °C °C

8.8.223 220 15:00 5:04 18:21 675 509 165 44.0 37.2 59.1 5.6 46.6 36.6

day of time sun sun Gact Sact Dact Ts Ta Tmrt PMV PET SET*

date year h:mm rise set W/m2 W/m2 W/m2 °C °C °C °C °C

8.8. 23 220 18:00 5:04 18:21 59 21 38 35.4 36.5 31.5 3.2 35.3 27.2

day of time sun sun Gact Sact Dact Ts Ta Tmrt PMV PET SET*

date year h:mm rise set W/m2 W/m2 W/m2 °C °C °C °C °C

8.8.23 220 21:00 5:04 18:21 0 0 0 31.0 33.0 22.5 1.3 27.8 21.2

day of time sun sun Gact Sact Dact Ts Ta Tmrt PMV PET SET*

date year h:mm rise set W/m2 W/m2 W/m2 °C °C °C °C °C

8.8.23 220 0:00 5:04 18:21 0 0 0 30.0 32.7 21.9 1.1 27.2 21.0

--------------------------------------------------------------------------------------------------------------------------------------------------

day of time sun sun Gact Sact Dact Ts Ta Tmrt PMV PET SET*

date year h:mm rise set W/m2 W/m2 W/m2 °C °C °C °C °C

15.8. 23 227 3:00 5:08 18:14 0 0 0 19.9 22.7 9.7 -3.0 15.9 5.5

day of time sun sun Gact Sact Dact Ts Ta Tmrt PMV PET SET*

date year h:mm rise set W/m2 W/m2 W/m2 °C °C °C °C °C

15.8. 23 227 6:00 5:08 18:14 44 15 29 18.7 21.0 12.8 -3.1 15.6 6.6

day of time sun sun Gact Sact Dact Ts Ta Tmrt PMV PET SET*

date year h:mm rise set W/m2 W/m2 W/m2 °C °C °C °C °C

15.8. 23 227 9:00 5:08 18:14 663 499 164 34.3 23.8 48.7 0.3 27.5 22.8

day of time sun sun Gact Sact Dact Ts Ta Tmrt PMV PET SET*

date year h:mm rise set W/m2 W/m2 W/m2 °C °C °C °C °C

15.8. 23 227 12:00 5:08 18:14 935 780 155 42.2 29.9 52.7 2.4 34.6 27.9

day of time sun sun Gact Sact Dact Ts Ta Tmrt PMV PET SET*

date year h:mm rise set W/m2 W/m2 W/m2 °C °C °C °C °C

15.8. 23 227 15:00 5:08 18:14 663 499 164 42.0 37.0 57.6 5.2 45.2 34.8

day of time sun sun Gact Sact Dact Ts Ta Tmrt PMV PET SET*

date year h:mm rise set W/m2 W/m2 W/m2 °C °C °C °C °C

15.8. 23 227 18:00 5:08 18:14 44 15 29 33.1 34.0 26.9 2.0 30.4 22.7

day of time sun sun Gact Sact Dact Ts Ta Tmrt PMV PET SET*

date year h:mm rise set W/m2 W/m2 W/m2 °C °C °C °C °C

15.8. 23 227 21:00 5:08 18:14 0 0 0 30.6 32.1 19.9 0.8 26.1 18.9

day of time sun sun Gact Sact Dact Ts Ta Tmrt PMV PET SET*

date year h:mm rise set W/m2 W/m2 W/m2 °C °C °C °C °C

15.8. 23 227 0:00 5:08 18:14 0 0 0 28.2 29.4 17.6 -0.4 23.2 14.1

__________________________________________________________________________________________

day of time sun sun Gact Sact Dact Ts Ta Tmrt PMV PET SET*

date year h:mm rise set W/m2 W/m2 W/m2 °C °C °C °C °C

22.8. 23 234 3:00 5:13 18:07 0 0 0 12.2 13.5 0.1 -7.7 6.0 -15.8

day of time sun sun Gact Sact Dact Ts Ta Tmrt PMV PET SET*

date year h:mm rise set W/m2 W/m2 W/m2 °C °C °C °C °C

22.8. 23 234 6:00 5:13 18:07 30 11 19 12.5 13.9 4.0 -7.0 7.1 -11.6

day of time sun sun Gact Sact Dact Ts Ta Tmrt PMV PET SET*

date year h:mm rise set W/m2 W/m2 W/m2 °C °C °C °C °C

22.8. 23 234 9:00 5:13 18:07 652 492 160 24.1 18.0 40.6 -3.5 17.1 7.3

day of time sun sun Gact Sact Dact Ts Ta Tmrt PMV PET SET*

date year h:mm rise set W/m2 W/m2 W/m2 °C °C °C °C °C

22.8. 23 234 12:00 5:13 18:07 927 776 151 29.3 20.0 41.7 -2.6 19.1 10.3

day of time sun sun Gact Sact Dact Ts Ta Tmrt PMV PET SET*

date year h:mm rise set W/m2 W/m2 W/m2 °C °C °C °C °C

22.8. 23 234 15:00 5:13 18:07 652 492 160 32.4 26.5 47.5 0.3 26.2 20.9

day of time sun sun Gact Sact Dact Ts Ta Tmrt PMV PET SET*

date year h:mm rise set W/m2 W/m2 W/m2 °C °C °C °C °C

22.8. 23 234 18:00 5:13 18:07 30 11 19 28.2 29.2 20.4 -0.3 23.5 14.7

day of time sun sun Gact Sact Dact Ts Ta Tmrt PMV PET SET*

date year h:mm rise set W/m2 W/m2 W/m2 °C °C °C °C °C

22.8. 23 234 21:00 5:13 18:07 0 0 0 22.3 24.7 11.8 -2.3 18.0 7.5

day of time sun sun Gact Sact Dact Ts Ta Tmrt PMV PET SET*

date year h:mm rise set W/m2 W/m2 W/m2 °C °C °C °C °C

22.8. 23 234 0:00 5:13 18:07 0 0 0 20.0 23.4 10.1 -2.6 16.6 7.6

__________________________________________________________________________________________

day of time sun sun Gact Sact Dact Ts Ta Tmrt PMV PET SET*

date year h:mm rise set W/m2 W/m2 W/m2 °C °C °C °C °C

1.9. 22 244 3:00 5:18 17:55 0 0 0 11.8 15.2 1.7 -5.4 8.2 -3.8

day of time sun sun Gact Sact Dact Ts Ta Tmrt PMV PET SET*

date year h:mm rise set W/m2 W/m2 W/m2 °C °C °C °C °C

1.9. 22 244 6:00 5:18 17:55 12 5 7 14.6 16.4 5.7 -5.6 9.8 -5.5

day of time sun sun Gact Sact Dact Ts Ta Tmrt PMV PET SET*

date year h:mm rise set W/m2 W/m2 W/m2 °C °C °C °C °C

1.9. 22 244 9:00 5:18 17:55 631 476 154 28.1 17.9 43.6 -2.1 20.8 14.9

day of time sun sun Gact Sact Dact Ts Ta Tmrt PMV PET SET*

date year h:mm rise set W/m2 W/m2 W/m2 °C °C °C °C °C

1.9. 22 244 12:00 5:18 17:55 910 765 145 36.6 23.8 48.6 -0.1 25.8 21.2

day of time sun sun Gact Sact Dact Ts Ta Tmrt PMV PET SET*

date year h:mm rise set W/m2 W/m2 W/m2 °C °C °C °C °C

1.9. 22 244 15:00 5:18 17:55 631 476 154 41.6 30.0 53.6 3.1 38.1 31.7

day of time sun sun Gact Sact Dact Ts Ta Tmrt PMV PET SET*

date year h:mm rise set W/m2 W/m2 W/m2 °C °C °C °C °C

1.9. 22 244 18:00 5:18 17:55 12 5 7 28.3 30.9 19.0 0.2 24.8 17.9

day of time sun sun Gact Sact Dact Ts Ta Tmrt PMV PET SET*

date year h:mm rise set W/m2 W/m2 W/m2 °C °C °C °C °C

1.9. 22 244 21:00 5:18 17:55 0 0 0 24.4 28.0 14.7 -1.0 21.3 13.9

day of time sun sun Gact Sact Dact Ts Ta Tmrt PMV PET SET*

date year h:mm rise set W/m2 W/m2 W/m2 °C °C °C °C °C

1.9. 22 244 0:00 5:18 17:55 0 0 0 21.7 25.0 12.9 -1.9 18.6 10.7
